# Vulvar Epidermolytic Hyperkeratosis: A Comprehensive Systematic Review of Case Reports and Series

**DOI:** 10.3390/jcm14010094

**Published:** 2024-12-27

**Authors:** Miruna Ioana Cristescu, Elena Codruța Cozma, Cristina Beiu, Irina Tudose, Selda Ali, Anca Bobircă, Liliana Gabriela Popa

**Affiliations:** 1 Clinic of Dermatology, Elias Emergency University Hospital, 011461 Bucharest, Romania; miruna-ioana.cristescu@rez.umfcd.ro (M.I.C.); elena-codruta.dobrica@rez.umfcd.ro (E.C.C.); 2 Department of Oncologic Dermatology-Elias Emergency University Hospital, Carol Davila University of Medicine and Pharmacy, 020021 Bucharest, Romania; liliana.popa@umfcd.ro; 3 Pathology Department, Elias Emergency University Hospital, 011461 Bucharest, Romania; irina.tudose@spitalul-elias.ro; 4 Allergology Department, Carol Davila University of Medicine and Pharmacy, 020021 Bucharest, Romania; selda.ali@umfcd.ro; 5 Department of Internal Medicine and Rheumatology, “Dr. Ion Cantacuzino” Clinical Hospital, Carol Davila University of Medicine and Pharmacy, 020021 Bucharest, Romania; anca.bobirca@umfcd.ro

**Keywords:** epidermolytic hyperkeratosis, multiple epidermolytic acanthoma, vulvar

## Abstract

**Background**: Vulvar epidermolytic hyperkeratosis (EHK) is an exceedingly rare dermatological condition, often presenting as solitary or multiple lesions in the vulvar region. Due to its clinical resemblance to other vulvar disorders, such as condyloma acuminatum, Bowenoid papulosis, and squamous cell carcinoma, vulvar EHK poses significant diagnostic challenges. While individual case reports and small case series have documented instances of vulvar EHK, comprehensive studies systematically consolidating the clinical, histopathological, and therapeutic aspects of this condition remain lacking. **Objectives**: To address this gap, this systematic review consolidates all available case reports and case series on vulvar EHK. The review aims to provide a comprehensive analysis of clinical presentations, histopathological features, diagnostic challenges, treatment approaches, and patient outcomes. **Methods**: We conducted a systematic review following the PRISMA guidelines. We searched multiple databases (PubMed, Web of Science, Scopus) for studies published up to 30 September 2024. Only case reports and case series with histopathologically confirmed vulvar EHK were included, as no higher-level studies (e.g., randomized controlled trials or cohort studies) were available due to the rarity of this condition. Exclusion criteria were male cases, oral EHK or other unrelated conditions, and literature reviews. We extracted and analyzed data on: patient demographics, time to diagnosis, anatomical distribution, clinical presentation, associated symptoms, histopathological features, patient history, risk factors, HPV status, treatment, and outcomes. Risk of bias was assessed using the CARE checklist and JBI Checklist for Case Series. Additionally, original clinical and histopathological images from our department were included to enhance the review. **Results**: A total of 19 studies, encompassing 30 cases of histopathologically confirmed vulvar EHK, were identified. Most cases presented with hyperkeratotic plaques and papules localized on the labia majora. Histopathological analysis consistently revealed hyperkeratosis, acanthosis, and vacuolar degeneration in the granular and spinous layers. Misdiagnosis was common, with lesions frequently mistaken for condyloma acuminatum or other vulvar neoplasms. Conservative management, including observation and topical therapies, was associated with disease stability in asymptomatic cases, while surgical excision demonstrated complete remission in all cases where it was employed. The rarity of vulvar EHK and reliance on case reports and series limit the generalizability of findings. **Conclusions**: Vulvar EHK is often misdiagnosed due to its similarity to malignancies and sexually transmitted infections. This review, the first of its kind, highlights the importance of prompt histopathological diagnosis to avoid the psychological impact of a cancer or sexually transmitted disease diagnosis and unnecessary, distressing, or aggressive treatments. Further research is needed to explore the role of HPV in vulvar EHK and to establish standardized diagnostic and treatment guidelines.

## 1. Introduction

Epidermolytic hyperkeratosis (EHK) is a histopathological pattern rather than a singular clinical entity. The hallmark histological features of EHK include hyperkeratosis, hypergranulosis, acanthosis, papillomatosis, vacuolar degeneration of keratinocytes in the granular and spinous layers, and occasionally reticular degeneration presenting as a net-like fragmentation of the granular layer [1].

This pattern can be observed in a variety of dermatological conditions, both congenital and acquired, which has led to some terminological confusion. In genetic disorders, EHK is the hallmark of epidermolytic ichthyosis (EI), formerly known as bullous congenital ichthyosiform erythroderma (BCIE) [2]. EI is an autosomal dominant condition caused by germline mutations in the keratin 1 (KRT1) or keratin 10 (KRT10) genes. These mutations result in generalized skin involvement, presenting at birth with generalized blistering and erythroderma, later progressing to chronic hyperkeratosis and scaling. The term EI has replaced BCIE to more accurately reflect the genetic and clinical features of this lifelong ichthyotic disorder [3].

Small, localized areas of EHK can occasionally be observed in other dermatological conditions, such as epidermal nevi, seborrheic keratosis, and viral warts, often detected incidentally during histopathological examination [1,4,5]. These foci are thought to result from post-zygotic mosaicism involving mutations in the KRT1 or KRT10 genes within a specific stem cell population.

The term epidermolytic acanthoma refers to isolated, benign, and acquired lesions exhibiting the characteristic histopathological features of EHK. These lesions typically present as solitary papules or plaques with normal surrounding skin and are unrelated to genetic disorders. Clinically, they often resemble seborrheic keratoses or warts, making diagnosis reliant on histopathological findings, which show EHK confined to the lesion itself [6]. Vulvar involvement is exceedingly rare, with only sporadic cases documented in the medical literature. In the context of vulvar lesions, the terms vulvar epidermolytic acanthoma and vulvar EHK are used interchangeably in the literature. For consistency, we will refer to these lesions as vulvar EHK throughout this paper.

When EHK involves the vulgar region, its clinical resemblance to conditions such as condyloma acuminatum, Bowenoid papulosis, and squamous cell carcinoma [6,7,8] poses significant diagnostic challenges, often leading to misdiagnosis, unnecessary treatments, patient anxiety, and delayed appropriate management. Although individual case reports and small case series have documented instances of vulvar EHK, the current literature is fragmented and lacks systematic consolidation of clinical, histopathological, and therapeutic data. This systematic review was conducted to address these gaps in knowledge. The primary objective is to provide a comprehensive analysis of all published case reports and case series on vulvar EHK, focusing on clinical presentations, histopathological features, diagnostic challenges, treatment modalities, and patient outcomes. Additionally, the review aims to identify trends in misdiagnosis, evaluate the role of human papillomavirus (HPV) in the pathogenesis of vulvar EHK, and explore the variability in therapeutic responses. By systematically reviewing the available evidence, this study seeks to enhance diagnostic accuracy, guide effective treatment strategies, and provide a foundation for future research in this field.

## 2. Materials and Methods

This systematic review was performed according to ”The Preferred Reporting Items for Systematic reviews and Meta-Analyses (PRISMA)” guidelines [9]. The investigators performed an advanced search of the medical literature across multiple databases (PubMed/Medline, Web of Science, and SCOPUS) from inception until 30 September 2024. The search strategy incorporated a combination of MeSH terms and free-text terms: “vulvar epidermolytic acanthoma”, “genital epidermolytic acanthoma”, “vulvar epidermolytic hyperkeratosis”, and “genital epidermolytic hyperkeratosis”. The search strategy was tailored to each database and included Boolean operators to optimize sensitivity and specificity. This study is not registered in any database.

Several inclusion criteria had to be met for articles to be included in this systematic review, namely: (1) the papers were case reports or case series reporting histopathologically confirmed vulvar EHK. Given the rarity of EHK, no higher-level studies (e.g., randomized controlled trials or cohort studies) were available; (2) the selected papers were presented in a language spoken by the authors (English, French, or Romanian); (3) the full text of the case reports/case series was accessible.

Exclusion criteria included the following: (1) the papers reporting cases of genital EHK in male patients; (2) the papers describing oral EHK or other unrelated conditions; (3) the papers that were literature reviews.

The process of article selection involved an initial screening of titles from the search results based on the specified keyword combinations. Each record was independently screened by two reviewers to ensure adherence to the inclusion and exclusion criteria. Following this preliminary scan, abstracts and full-text articles of the identified studies were then thoroughly examined, with two reviewers independently assessing each report. The process was documented using a PRISMA flow diagram.

A structured database was created in Microsoft Excel to systematically record the following key details from each selected paper: first author name, year of publication, the age of the patients at diagnosis, time to diagnosis, anatomical location of the lesions, clinical characteristics, associated symptoms, histopathological findings, the presence of presumed risk factors for the development of EHK, status of HPV testing, prior misdiagnoses, as well as the recommended treatment and outcome. Furthermore, two independent reviewers assessed the quality of the included studies using the CARE checklist for case reports and the Joanna Briggs Institute (JBI) Critical Appraisal Checklist for Case Series.

Given the rarity of EHK and the heterogeneity of the reported cases, we employed a narrative synthesis. Key findings were summarized descriptively in Table 1. The decision to use narrative synthesis was based on the qualitative nature of the data and the absence of homogeneity required for meta-analysis.

In addition to the systematic review, we have included clinical and histopathological images from the Dermatology and Pathology departments at Elias Emergency University Hospital. These images illustrate key diagnostic features of vulvar EHK and were collected in compliance with ethical guidelines and with informed consent from the patients.

## 3. Results

### 3.1. Literature Search Results

**Figure 1:**
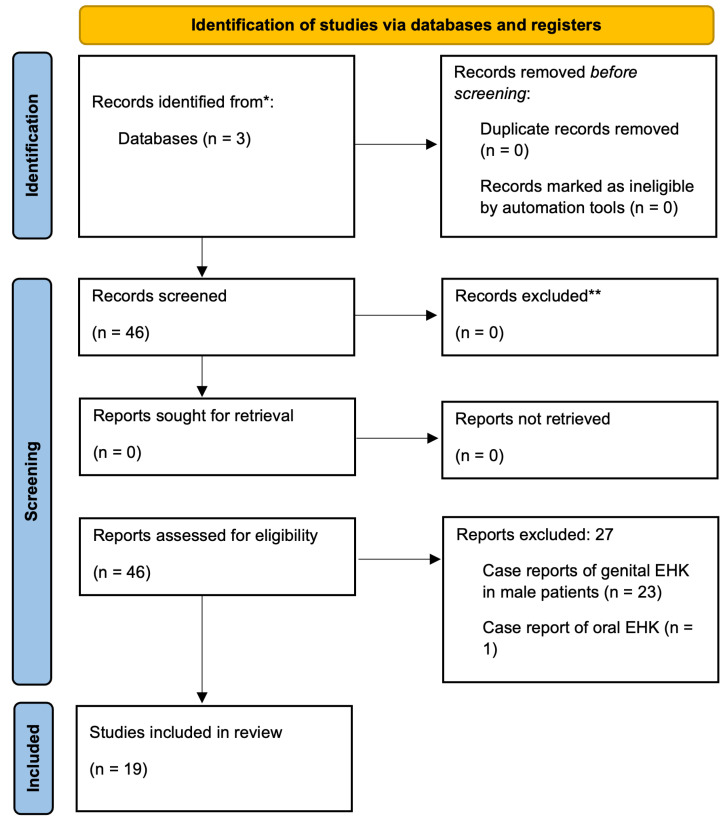
PRISMA flow diagram: Nineteen relevant publications were identified through database searching and were all included in the qualitative and quantitative synthesis. * records excluded based on title and abstract screening for not meeting inclusion criteria; ** records excluded during full-text review due to irrelevance or lack of histopathological confirmation.

The initial search across the databases yielded 46 records. After screening and applying eligibility criteria, 27 articles were excluded: 23 involved cases of genital EHK in male patients, one described oral EHK, two pertained to unrelated conditions, and one was a literature review. Ultimately, 19 studies reporting a total of 30 cases of EHK in the anogenital region of female patients were included in the review. Figure 1 presents a PRISMA flow diagram outlining the selection process for the articles included in this systematic review. The key characteristics of these studies are summarized in Table 1 and are discussed in detail below.

### 3.2. Patient Demographics and Diagnostic Timeline

The mean age of female patients with genital EHK was 58.4 ± 14.7 years, with the youngest patient diagnosed at the age of 24 and the eldest at the age of 91. The duration of the condition before diagnosis ranged from 1 week to over 30 years, with a mean duration of 7 years.

### 3.3. Anatomical Distribution and Clinical Presentation

The most common location was the labia majora (26 cases, 87%). EHK located on the buttocks, perineum, and perianal area was also reported. The vaginal wall was affected in only 2 cases (6.7%); in one case, the mucosal lesions were accompanied by vulvar EHK. The clinical manifestations of vulvar EHK varied across cases: three cases manifested as solitary epidermolytic acanthoma, one case as linear EHK of the vulva, extending to the perineum and ipsilateral thigh [12], and the rest of the patients presented with multiple verrucous or hyperkeratotic papules, usually white or hypopigmented, often with a tendency to coalesce into plaques. Twenty-one of the thirty patients (70%) complained of associated symptoms. The most frequent accompanying symptom was pruritus, reported by 12 patients (57%). Other complaints were pain and a burning sensation. It is noteworthy that in six patients (30%), the lesions were completely asymptomatic.

### 3.4. Histopathological Findings

The histopathological analysis of vulvar EHK revealed consistent features across the reviewed cases. Hyperkeratosis was universally present in all cases (100%). Acanthosis, indicating epidermal thickening, was observed in 80% of cases, while papillomatosis, characterized by a wavy surface architecture, was noted in 73%. Hypergranulosis, reflecting thickening of the granular layer, was identified in 67% of cases. Vacuolar degeneration of keratinocytes, a hallmark feature, was observed in 93% of cases. This finding, localized predominantly in the granular and spinous layers, was described in various ways by the authors. Common descriptions included cytoplasmic clearing resulting in granular degeneration and perinuclear clear zones, with intra- and intercellular edema. Prominent basophilic aggregated keratohyalin granules, often clumped or irregular, described as globules or streaks, were frequently noted alongside eosinophilic inclusion bodies, which appeared as rounded “globoid” structures or streaks in the granular and spinous layers. Three authors described vacuolar degeneration as dyskeratotic changes [11,12,18]. Reticular degeneration was described in only 16% of the cases. One case described foci of EHK within a vulvar basal cell carcinoma (BCC) in a patient with a history of intravaginal condyloma acuminatum and vaginal intraepithelial neoplasia 3 (VAIN 3), with HPV type 42 detected in both the condyloma and VAIN 3 specimens but not in the BCC associated with EHK [15].

### 3.5. Patient History, Risk Factors, and HPV Testing

A relevant family history was identified in only one patient presenting with vulvar and oral EHK, with similar oral lesions having been observed in the patient’s brother and father. None of the patients reported local trauma as a possible triggering factor. Only one patient suffered from immunosuppression due to Waldenstrom’s macroglobulinemia [9]. The patients did not present concomitant genital dermatoses, except for one patient suffering from genital lichen sclerosus and one patient from vulvar psoriasis. EHK was incidentally diagnosed in one patient undergoing surgery for vulvar BCC. HPV testing was performed in 17 patients and was negative in 16 patients. HPV type 42 was identified in one patient, previously diagnosed with intravaginal condyloma acuminatum and vaginal intraepithelial neoplasia (VAIN) 3, but the viral DNA was not detected in the epidermolytic acanthoma [15].

### 3.6. Previous Misdiagnosis and Treatment

Previous misdiagnosis was very common, the lesions having been confused especially with condyloma acuminatum and Bowenoid papulosis, but also with common warts, seborrheic keratoses, molluscum contagiosum, benign intraepithelial dyskeratosis, nevi, and squamous cell carcinoma.

Treatment strategies across the included cases demonstrated diverse approaches with varying levels of effectiveness. Excision was used in two cases (6.7%), both of which resulted in complete remission, indicating a 100% success rate for surgical intervention in the cases reviewed. In contrast, conservative management with no treatment was documented in three cases (10%), all of which remained stable, suggesting this approach may be appropriate for asymptomatic or less severe presentations. Topical treatments also showed promising results. Topical pimecrolimus achieved clinical remission in one case (3.3%), while topical lactic acid and a combination therapy of 0.1% estriol, 2% miconazole, and 1% hydrocortisone resulted in clinical improvement in two cases (6.7%). Additionally, topical tacrolimus with barrier creams led to complete remission of symptoms and partial resolution of skin lesions in one case (3.3%), highlighting the potential efficacy of immunomodulatory treatments. In contrast, the use of high-potency corticosteroids combined with topical estrogen provided no clinical benefit in one case (3.3%), whereas a regimen of corticosteroids for two months, followed by as-needed application, resulted in complete remission of pruritus and partial improvement of skin lesions within one month in one case (3.3%). Overall, nine cases (30%) lacked detailed information on treatment (marked as “UA”), and four cases (13.3%) employed a “watch and wait” strategy or conservative therapies like cryotherapy, which led to stable disease.

**Figure 2:**
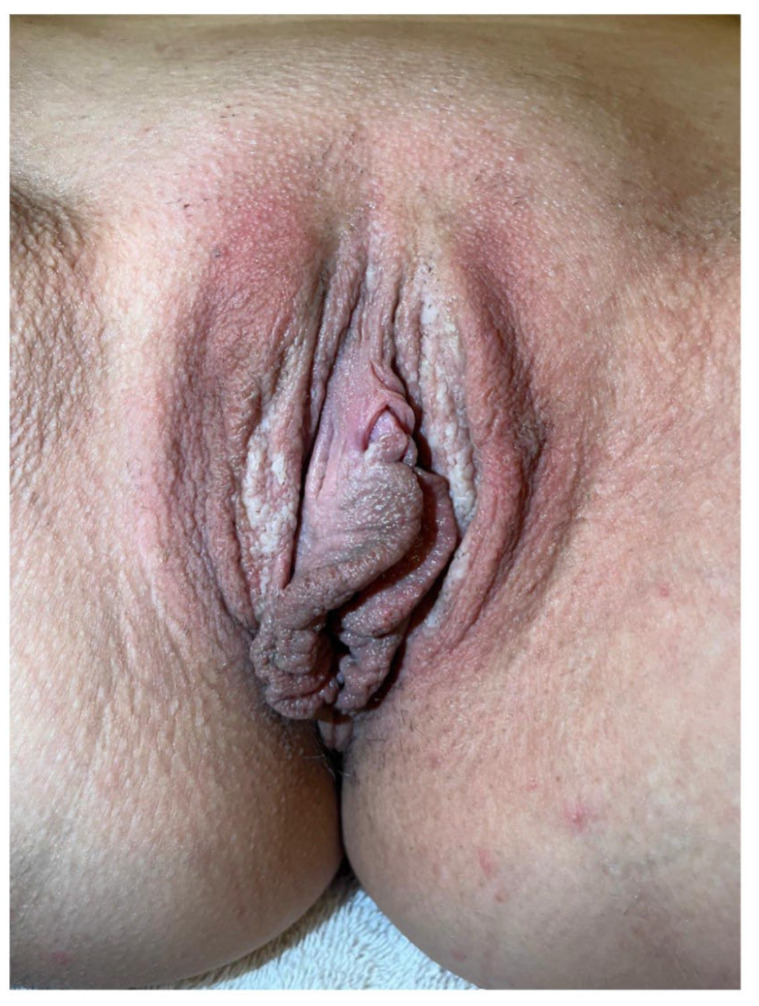
Representative clinical image of a patient with vulvar EHK, showing multiple grey-colored, hyperkeratotic papules coalescing into plaques located bilaterally on the labia majora (clinical photograph taken at Elias Emergency University Hospital, Bucharest, Romania).

We wish to add our own experience to the existing evidence by describing the case of a 25-year-old female with no significant family and personal medical history, who presented to our clinic for the presence of multiple grey-colored, hyperkeratotic papules coalescing into plaques located bilaterally on the labia majora. The surface of the plaques displayed significant fragility, exfoliating upon minor trauma (Figure 2). The patient complained of local discomfort and occasional pruritus. The lesions appeared 3 years previously and followed an unpredictable course, with long periods of remission and exacerbations in the absence of an evident trigger. The patient underwent an annual gynecological examination and was recently diagnosed with cervical HPV 6 infection. Over the previous 3 years, the vulvar lesions were diagnosed as chronic dermatitis and treated with multiple short courses of topical corticosteroids and daily use of emollients. As no clinical improvement was achieved, clinical suspicion of condyloma acuminatum was raised, and the patient was recommended local keratolytic treatment with topical salicylic acid, as well as topical podophyllotoxin and sinecatechins, which initially led to a regression of the lesions. A few months after the discontinuation of the mentioned treatment, the lesions recurred, and the patient was referred to our clinic. The patient had ceased all topical treatments 3 months before presentation in our department. Based on the clinical examination, EHK was considered. The rest of the physical examination did not reveal pathologic findings.

**Figure 3:**
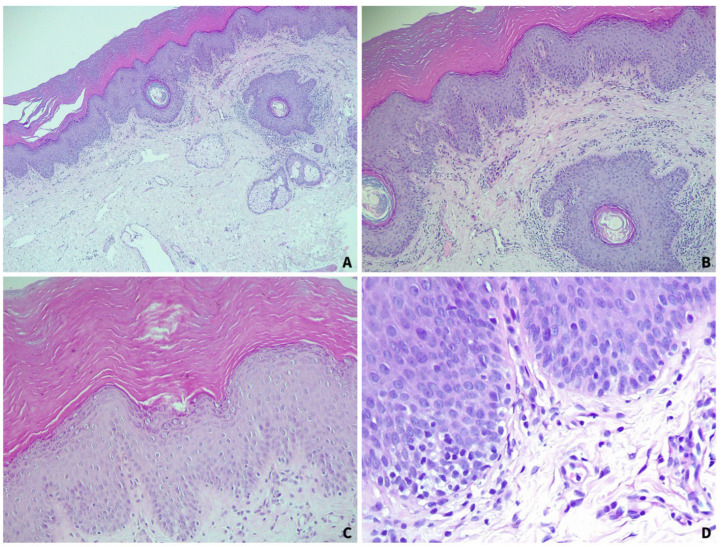
Hematoxylin–eosin stain showing hyperorthokeratosis, focal hypergranulosis, and the presence of irregular keratohyalin granules, along with vacuolar degeneration of the granular and superficial spinous layers, and a minimal lymphocytic inflammatory infiltrate in the superficial dermis ((**A**): 50×; (**B**): 100×; (**C**): 200×; (**D**): 400×) (histopathology images from the Pathology Department at Elias Emergency University Hospital, Bucharest, Romania).

In order to confirm the clinical diagnosis, a biopsy of the vulvar lesions was performed. The histopathological examination revealed hyperorthokeratosis, focal hypergranulosis, and the presence of irregular keratohyalin granules, along with vacuolar degeneration of the granular and superficial spinous layers. A minimal inflammatory infiltrate predominantly composed of lymphocytes and plasma cells was observed in the superficial dermis, with minimal lymphocytic intraepithelial exocytosis (Figure 3).

The patient was informed about the benign nature of the condition and was recommended mild keratolytic creams and emollients.

## 4. Discussion

Epidermolytic hyperkeratosis is a histological reaction pattern first described by Brocq in 1902 in a case of congenital bullous ichthyosiform erythroderma and subsequently observed in a large array of skin lesions, as previously mentioned [13]. It was not until 1970 that solitary epidermolytic acanthoma was described and denominated by Shapiro and Baraf [24]. Three years later, Hirone and Fukushiro reported a case of multiple disseminated epidermolytic acanthoma [25]. EHK of the vulvar region is exceedingly rare, usually diagnosed incidentally, upon histopathological examination of biopsy specimens for other vulvar conditions [26].

EHK incidence is not known, the condition being frequently overlooked or misdiagnosed. The estimated incidence of genital solitary epidermolytic acanthoma varies greatly between studies, ranging from <1 to 8/100,000 cases [5,6]. Chan et al. examined 183 vulvar biopsy specimens obtained from non-neoplastic, non-infectious lesions and identified the EHK pattern in only one specimen [26].

The etiopathogenesis of the disease is unclear. The clinical and histological resemblance between EHK and inherited ichthyoses point to a possible role of functional mutations in genes encoding KRT1 and KRT10, which are essential for epidermal differentiation in the spinous layer. In normal keratinocytes, KRT1 and KRT10 form intermediate filaments that anchor to desmosomes, ensuring cellular cohesion and epidermal integrity [27]. Mutations in these genes lead to amino acid substitutions, resulting in misfolded keratin aggregates that impair the keratinocyte cytoskeleton and cause cellular lysis—the hallmark of vacuolar degeneration observed histologically [27].

The results of the two studies that have assessed the expression of KRT1 and KRT10 genes in EHK remain contradictory. While Cohen et al. detected reduced expression of the two genes [28], Egozi et al. did not identify KRT1 and KRT10 mutations in their patient [18]. This inconsistency may reflect heterogeneity in the disease’s genetic background or limitations in genetic testing methods. Additionally, mutations in keratin 4 (KRT4) and keratin 13 (KRT13), keratins expressed in oral and genital mucosae, have been proposed as potential contributors in lesions involving these sites [11,20,28]. However, none of the reported cases of genital EHK arising in female patients had a family history of ichthyoses or other genodermatoses, suggesting that these lesions may arise from post-zygotic mosaic mutations rather than germline inheritance. The personal and family history of our patient was also unremarkable.

Viral infections, trauma, and immunosuppression were suspected as potential triggers for genital EHK [29]. However, except for Jung et al., who reported HPV 16 positivity in a case of EHK of the scrotum [30], none of the studies performed so far confirmed the implication of HPV infection in the pathogenesis of the disease. The largest such study, conducted by Kazlouskaya et al., investigated the presence of HPV in 64 EA biopsy specimens using in situ hybridization but found no evidence of genital HPV infection [6]. Upon reviewing the published data regarding female genital EHK, we only found evidence of genital HPV infection in one case, in which HPV DNA was detected in specimens collected from lesions of intravaginal condyloma acuminatum and VAIN 3 but not from biopsy specimens obtained from the vulvar EHK [15], rendering the causal relationship unlikely. Our patient was diagnosed with cervical HPV 6 infection long after the appearance of the skin lesions. They were misdiagnosed as condyloma acuminatum and treated accordingly but proved refractory to topical treatment with keratolytics, podophyllotoxin, and sinecatechins. The histopathological examination showed no changes suggestive of HPV infection. Therefore, the connection between HPV 6 infection and EHK cannot be supported in our case.

No history of local trauma was reported by the patients diagnosed with vulvar EHK. Our patient also denied any traumatization of the region and did not show signs suggestive of grating. Additionally, all reported cases described immunocompetent patients, except one patient suffering from Waldenstrom macroglobulinemia [11].

Clinically, vulvar EHK generally presents as well-defined, skin-colored, white or gray papules, with a tendency to coalesce into one or multiple plaques. The lesions are often asymptomatic but may be accompanied by pruritus, pain, or local discomfort [4]. Atypical clinical forms of EHK have been described, such as linear EHK [29] and exophytic keratotic nodules [8], often confused with other inflammatory disorders or tumors. The clinical differential diagnoses include infectious diseases like condyloma acuminatum, bowenoid papulosis, molluscum contagiosum, inflammatory skin diseases such as chronic dermatitis and lichen planus, and benign and malignant tumors like verruca vulgaris or plana, white sponge nevus, or squamous cell carcinoma [4,6]. Differentiation of linear EHK from verrucous epidermal nevus, which can also have an adult onset, and zosteriform Darier’s disease is challenging [20,29].

The definite diagnosis of vulvar EHK is based on histopathological examination and requires the presence of typical changes in more than 50% of the lesion [21]. The characteristic features of EHK include hyperkeratosis, often with a mixture of orthokeratosis and focal parakeratosis, hypergranulosis, acanthosis, papillomatosis, and vacuolar degeneration of keratinocytes in the granular and spinous layers. Reticular degeneration, which refers to a fragmented, net-like appearance primarily in the granular layer, may also be observed, although it is less prominent compared to vacuolar degeneration [1].

Vacuolar degeneration is characterized by cytoplasmic clearing in keratinocytes due to disrupted keratin filament organization. This creates the appearance of the epidermis “falling apart” or “lysing”, although it is merely an artifact rather than true cellular destruction. It manifests as perinuclear pale zones or eosinophilic bands, accompanied by eosinophilic globules (misfolded keratin filaments) and basophilic globules (keratohyalin granules). These changes give keratinocytes an “empty” cytoplasmic appearance, often surrounded by amphophilic material [6,20,31]. Intra- and intercellular edema are frequently observed in the suprabasal layers, further contributing to the vacuolated appearance of keratinocytes [6].

The histological findings in EHK may be described by some authors as dyskeratosis, as seen in three cases included in our review [11,12,18]. This likely stems from the eosinophilic cytoplasm, perinuclear halos, and cytoplasmic changes observed in EHK keratinocytes, which resemble features of dyskeratosis. However, these changes do not meet the classical definition of dyskeratosis seen in conditions such as Darier’s disease or Grover’s disease, where keratinocytes undergo abnormal premature keratinization below the granular layer, characterized by pyknotic nuclei, corps ronds, and grains, often accompanied by acantholysis [32]. Instead, in EHK, the keratinocytes exhibit vacuolar degeneration, resulting in cytoplasmic clearing due to disorganized keratin filaments and clumped keratohyalin granules while retaining nuclear integrity and cell adhesion.

This histological distinction is essential for understanding the pathology and differentiating EHK from other clinical mimics. Although clinically, EHK can be difficult to differentiate from condyloma acuminatum, histopathological differentiation from HPV-induced lesions is straightforward. Koilocytes, with their typical raisinoid nuclei and perinuclear clearing, are absent, while keratohyalin clumping and balloon degeneration characteristic for EHK are prominent changes. While bowenoid papulosis may present similar histological changes, such as alteration of the granular layer, keratinocytes atypia and loss of normal polarity are also present and aid in the distinction between the two disorders [12]. The typical basophilic intranuclear inclusions of molluscum contagiosum are absent [8]. Histopathological differentiation from white sponge nevus is based on the absence of cells with pyknotic nuclei and dense eosinophilic cytoplasm rims, and the presence of keratohyalin granules [11]. Additionally, EHK does not present nuclear atypia observed with HPV-induced vulvar intraepithelial neoplasia or acantholytic actinic keratosis [21].

Based on the findings of this systematic review, we propose the following diagnostic criteria for vulvar EHK: clinically, lesions present as well-defined, skin-colored, whitish, or gray papules, with or without coalescence into plaques, and may be asymptomatic or associated with pruritus, burning, or pain. Histopathologically, the diagnosis requires the presence of hyperkeratosis, hypergranulosis with clumped keratohyalin granules, and vacuolar degeneration of keratinocytes in the granular and spinous layers, often accompanied by eosinophilic inclusion bodies and basophilic granular deposits. Additional features include acanthosis and papillomatosis. Exclusion of other conditions such as condyloma acuminatum (absence of koilocytes), Bowenoid papulosis, or squamous cell carcinoma (absence of nuclear atypia), and molluscum contagiosum is essential for diagnosis. The presence of these histopathological changes in more than 50% of the lesion is required for confirmation, as per existing evidence. HPV testing is not mandatory but may be useful in ruling out viral-induced lesions when clinically suspected. These criteria aim to clarify the diagnostic process, reduce misdiagnosis, and guide appropriate management.

EHK follows a benign natural course, carrying no potential for malignant transformation [13]. Therefore, it does not impose intensification in clinical monitoring and gynecologic screening. Treatment is optional, depending on the patient’s symptomatology and preferences. The first-line therapy includes hypoallergenic emollients. The use of glycerin and mild keratolytic products containing urea, salicylic, lactic, or glycolic acid softens the lesion and keeps the area smooth and hydrated [13]. Topical anti-inflammatory agents, like low-potency corticosteroids or calcineurin inhibitors, usually control the associated pruritus [1]. Topical 5% imiquimod cream has also been reported as efficient in a case of scrotal EHK [33]. Local destructive methods, including surgical excision, curettage, cryotherapy, or electrodesiccation, may also be employed in selected cases, being more suitable for single lesions or nodular presentations [12,13]. Such procedures should be carefully performed in order to avoid slow-healing deep ulcerations and scarring. Procedures like CO_2_ laser or topical podophyllin applications are considered aggressive and may result in a large denuded area, which requires prolonged care given the particularities of the genital region [12].

The evidence included in this review is subject to several limitations. The small number of reported cases limits the generalizability of the findings and the ability to establish robust conclusions about risk factors, pathogenesis, or optimal treatment. Variability in the level of detail provided in case reports and case series, particularly regarding follow-up and treatment outcomes, reduces the comparability of data and introduces challenges in synthesizing findings. Furthermore, as most data derive from published case reports, there is an inherent risk of publication bias, with more unusual or severe cases likely to be overrepresented. These limitations are compounded by the lack of standardized diagnostic and therapeutic protocols, which further reduces confidence in the findings for treatment outcomes.

Despite these constraints, certain aspects of the evidence, such as the histopathological features of vulvar EHK, were consistent across cases, lending a higher degree of confidence to these specific observations. However, variability in treatment approaches and patient outcomes highlights critical gaps in the literature. Additionally, the heterogeneity in study design and reporting precluded the use of meta-analytical techniques, limiting the ability to provide quantitative estimates of treatment efficacy or risk factors. These limitations underscore the need for standardized reporting of future cases to enhance understanding of this rare condition.

The findings of this review have important implications for clinical practice, healthcare policy, and future research. Clinicians should include EHK in the differential diagnosis of hyperkeratotic vulvar lesions, particularly in postmenopausal women. Accurate diagnosis through biopsy is crucial to avoid unnecessary aggressive treatments or mismanagement. Raising awareness about EHK among dermatologists and gynecologists could enhance diagnostic accuracy and patient care.

The benign nature of EHK suggests that conservative management is appropriate for asymptomatic cases. For symptomatic patients, individualized treatment plans involving topical therapies or surgical excision should be considered. These approaches aim to balance effective symptom relief with minimal invasiveness. Vulvar EHK should be explicitly recognized in dermatological and gynecological guidelines as a distinct differential diagnosis. Guidelines should include recommendations for histopathological confirmation and tailored treatment strategies to improve consistency in clinical practice and patient outcomes.

Several areas require further investigation to advance the understanding and management of EHK: (1) Research should focus on elucidating the etiology of EHK, particularly the role of keratin mutations and other potential genetic factors; (2) systematic collection of clinical, histopathological, and therapeutic data through prospective studies or a case registry could provide more robust evidence to guide practice; (3) evaluating the long-term outcomes and recurrence rates associated with different treatment modalities is essential to establish evidence-based management protocols.

By addressing these areas, future research can help refine diagnostic and therapeutic approaches, contributing to improved care for patients with vulvar EHK.

## 5. Conclusions

Due to its rarity, vulvar EHK is usually misdiagnosed, being most often confused with condyloma acuminatum, Bowenoid papulosis, common warts, or squamous cell carcinoma. Empiric treatment is generally recommended in these cases. Clinicians should consider EHK in the differential diagnosis of hyperkeratotic genital lesions and perform biopsies whenever the suspicion is raised, as histopathological examination is indispensable for a definite diagnosis. Prompt diagnosis is essential in order to avoid the psychological impact of a cancer or sexually transmitted disease diagnosis and unnecessary, distressing, or aggressive treatments. The management of EHK depends on the clinical presentation and the patient’s preference. As most patients are asymptomatic or complain of mild pruritus and the course of the disease is benign, reassurance of the patient and the use of bland emollients, barrier creams, and mild topical anti-inflammatory products generally suffice.

## Figures and Tables

**Table 1:** Vulvar EHK cases reported in the medical literature.

Publication	Age at Diagnosis (Years)	Time Until Diagnosis	Location	Clinical Findings	Associated Symptoms	Histopathology Findings	Relevant History and Risk Factors	HPV Testing	Previous Misdiagnosis	Treatment	Outcome
De Coninck et al., 1986 [10]	62	Several years	Left LM	Multiple verrucous light brown papules	Pruritus	Hyperkeratosis; acanthosis; large, irregularly shaped keratohyalin bodies; granular degeneration; premature keratinization; marked inter- and intracellular edema.	No	No	UA	UA	UA
Quinn et al., 1997 [11]	75	1 year	Vulva	Single 2/3 mm papule	NAS	Hyperkeratosis; acanthosis; papillomatosis; hypergranulosis; perinuclear clear zones in the suprabasilar epithelium; keratohyalin clumping; dyskeratosis	Immunosuppression due to Waldenstrom macroglobulinemia	No	UA	Excision	Remission
	40	UA	Vaginal wall	White HK plaque	NAS		The patient, her brother, and her father presented oral white, hyperkeratotic plaques	Negative IHC	Benign intraepithelial dyskeratosis (association with oral lesions)	UA	UA
Swann et al., 2003 [12]	58	2 years	Mons pubis, LM, Lm	Multiple HK papules of 4–8 mm	Pruritus Pain	Hyperkeratotic papillomatosis; epidermal acanthosis; hypergranulosis; perinuclear clear zones; clumping of granular keratohyalin; dyskeratosis resulting in intracellular eosinophilic inclusions	No	No	Bowenoid papulosis; Verruca	UA	UA
High et al., 2005 [13]	54	20 years	Vulva	Multiple white HK papules	NAS	Hyperkeratosis; acanthosis; papillomatosis; hypergranulosis; prominent reticular degeneration	No	Negative IHC	Condyloma acuminatum; Bowenoid papulosis	No treatment	Stable
Thomas et al., 2010 [14]	50	Early adulthood	Left LM, perineum, left thigh	Linear greyish-white verrucous plaque	Pruritus	Compact hyperkeratosis; localized acanthosis; minimal papillomatosis; deep invagination of the stratum corneum; vacuolization in the granular and spinous layers; abnormal accumulation of keratohyalin granules	No	No	Verruca; Condyloma acuminata; Localized Darier′s disease; Inflammatory verrucous epidermal naevus	UA	UA
Russell et al., 2010 [8]	69	1.5 months	Left vulva	Multiple pale, pearly, warty lesions of 3–4 mm	Pruritus Burning sensation	Marked hyperkeratosis; acanthotic granular and spinous layers; aggregated eosinophilic keratohyalin with peri-nuclear haloes in the spinous and granular layers; rounded eosinophilic “globoid” bodies in the stratum corneum	No	No	UA	0.1% estriol, 2% miconazole, and 1% hydrocortisone	Clinical improvement
Kazlouskaya et al., 2013 [6]	UA	UA	Labia	Sessile, tan or brownish isolated papules or exophytic, wart-like papules	UA	Epidermolytic hyperkeratosis, hyperkeratosis in the stratum corneum, occasionally orthokeratosis; cup-shaped configuration with papillomatous base; eosinophilic and basophilic inclusions as globules and streaks in the stratum corneum	UA	Negative ISH	Condylomata; Molluscum contagiosum; Nevus Squamous cell carcinoma	UA	UA
Kacerovska et al., 2014 [15]	79	UA	Right LM	Solitary whitish lesion with central desquamation	UA	Histologically diagnosed as BCC with multiple foci of EHK	Concomitant BCC, intravaginal condyloma acuminatum and VAIN 3	HPV 42 + in intravaginal condyloma acuminatum and VAIN 3, but—in EA and BCC	UA	Excision	Remission
Hijazi et al., 2015 [16]	31	2 years	Left LM	Multiple hypopigmented flat papules	Pruritus	Compact hyperkeratosis; cup-shaped invagination of the epidermis; mild papillomatosis; hypergranulosis; vacuolar degeneration of keratinocytes in the spinous and granular layers; amorphous eosinophilic trichohyaline-like granules	No	No	UA	Topical pimecrolimus	Clinical remission
Fletcher et al., 2016 [17]	59	Several months	Bilateral LM	Multiple HK papules	NAS	Hyperkeratosis; papillomatosis; hypergranulosis with keratohyaline aggregates; multifocal vacuolization of suprabasilar keratinocytes	No	No	UA	Topical lactic acid	Clinical improvement
Egozi-Reinman et al., 2016 [18]	47	UA	External genital area	Multiple whitish or skin colored papules	NAS	Hyperkeratosis; acanthosis; epidermolytic and dyskeratotic changes in the upper spinous and granular cell layers	No	Negative ISH for HPV DNA	UA	UA	UA
Moulonguet et al., 2017 [19]	50	UA	LM, Lm bilateral	Multiple verrucous papules	Pain Burning sensation	Compact orthokeratotic hyperkeratosis; acanthosis; reticular deposits and irregular keratohyalin granules in the spinous and granular layers; eosinophilic bodies present within the cytoplasm	No	No	Condyloma acuminatum	Emollients	Improvement of symptoms, persistence of lesions
Lee and Wu, 2017 [20]	91	1 week	LM	Multiple whitish smooth papules	Pruritus	Compact hyperkeratosis; perinuclear vacuolization; reticular degeneration in the granular and upper spinous layers; irregular basophilic keratohyaline granules; eosinophilic inclusion bodies in the upper spinous layers	No	Negative PCR for high and low risk HPV	Condyloma acuminatum; Bowenoid papulosis	Watch and wait	Stable
	46	>1 month	LM	Multiple whitish smooth papules	Pruritus		No	Negative PCR for high and low risk HPV	Condyloma acuminatum	Cryotherapy	Stable
Iglesias-Plaza et al., 2018 [4]	61	2 months	Left LM	White HK plaque	NAS	Hyperkeratosis; parakeratosis; acanthosis; hypergranulosis; marked reticular degeneration	Genital lichen sclerosus	No	Squamous cell carcinoma	No treatment	Stable
Irwin et al., 2018 [7]	46	UA	Bilateral LM	Multiple white papules	Pruritus	Pronounced hyperkeratosis (orthokeratosis); acanthosis, papillomatosis; mild vacuolar changes in the surface squamous epithelium; granular layer thickening with prominent keratohyalin granules	Vulvar psoriasis	Negative IHC for HPV 16	Keratosis	Topical tacrolimus Barrier creams	Complete remission of symptoms and partial remission of skin lesions
	61	Several months	Bilateral LM	Multiple hyperkeratotic papules	Pruritus		No	Negative IHC for HPV 16	UA	UA	UA
Roy et al., 2020 [21]	75	UA	Butttock	Solitary papules	UA	UA	UA	HPV genotyping was performed in one patient and was negative	Inverted follicular keratosis; Verruca vulgaris Squamous cell carcinoma	UA	UA
	58		Labia						Verruca vulgaris		
	71		Labia						UA		
	61		Buttock						Nevus		
	72		Anus						UA		
	46		Labia						Seborrheic keratosis; Verruca		
	24		Labia						UA		
Dai et al., 2021 [1]	68	Several years	Right LM, focal involvement of the inner mucosa	Multiple whitish papules with variable confluence into small plaques	Pruritus	Hyperkeratosis; papillomatosis; hypergranulosis; epidermal pallor; vacuolar degeneration of the spinous and granular layer with course basophilic keratohyalin granules	No	No	Vulvar malignancy	High potency CST, Topical estrogen	No clinical benefit
Farahbakhsh et al., 2022 [22]	62	5 years	LM bilateral	Multiple hypopigmented verrucous papules	Pruritus	Marked hyperkeratosis; acanthosis; papillomatosis with cup-shaped lesion structure; vacuolization in the granular layer; prominent basophilic granules within the granular layer; subtle eosinophilic granules in both the granular and spinous layers	No	Negative IHC stains for high and low risk HPV	Common wart	No treatment	Stable
Sachedina et al., 2023 [23]	62	30 years	Vulva	Multiple clustered, grey/white, flat shiny papules	Pruritus	Marked hyperkeratosis; slight epidermal hyperplasia; hypergranulosis; vacuolar changes in the granular and spinous layers; prominent keratohyalin granules	No	No	UA	CST for 2 months, then as needed	Complete remission of pruritus and partial remission of skin lesions in 1 month

HPV—human papillomavirus; LM—labia majora; UA—unavailable information; NAS—no associated symptoms; HK—hyperkeratotic; IHC—immunohistochemistry; Lm—labia minora; ISH—in situ hybridization; VAIN 3—vaginal intraepithelial neoplasia 3; DNA—deoxyribonucleic acid; BCC—basal cell carcinoma; CST—topical corticosteroids; EHK—epidermolytic hyperkeratosis; EA—epidermolytic acanthoma.
